# Trib1 Is Overexpressed in Systemic Lupus Erythematosus, While It Regulates Immunoglobulin Production in Murine B Cells

**DOI:** 10.3389/fimmu.2018.00373

**Published:** 2018-03-15

**Authors:** Léa Simoni, Virginia Delgado, Julie Ruer-Laventie, Delphine Bouis, Anne Soley, Vincent Heyer, Isabelle Robert, Vincent Gies, Thierry Martin, Anne-Sophie Korganow, Bernardo Reina-San-Martin, Pauline Soulas-Sprauel

**Affiliations:** ^1^CNRS UPR 3572 “Immunopathology and Therapeutic Chemistry”/Laboratory of Excellence Medalis, Institute of Molecular and Cellular Biology (IBMC), Strasbourg, France; ^2^UFR Médecine, Université de Strasbourg, Strasbourg, France; ^3^Institut de Génétique et de Biologie Moléculaire et Cellulaire (IGBMC), Illkirch, France; ^4^Institut National de la Santé et de la Recherche Médicale (INSERM), U964, Illkirch, France; ^5^Centre National de la Recherche Scientifique (CNRS), UMR7104, Illkirch, France; ^6^Université de Strasbourg, Illkirch, France; ^7^Department of Clinical Immunology and Internal Medicine, National Reference Center for Autoimmune Diseases, Hôpitaux Universitaires de Strasbourg, Strasbourg, France; ^8^UFR Sciences pharmaceutiques, Université de Strasbourg, Illkirch-Graffenstaden, France

**Keywords:** lupus, B cells, Trib1, mouse model, Ig secretion, negative regulator

## Abstract

Systemic lupus erythematosus (SLE) is a severe and heterogeneous autoimmune disease with a complex genetic etiology, characterized by the production of various pathogenic autoantibodies, which participate in end-organ damages. The majority of human SLE occurs in adults as a polygenic disease, and clinical flares interspersed with silent phases of various lengths characterize the usual evolution of the disease in time. Trying to understand the mechanism of the different phenotypic traits of the disease, and considering the central role of B cells in SLE, we previously performed a detailed wide analysis of gene expression variation in B cells from quiescent SLE patients. This analysis pointed out an overexpression of *TRIB1*. TRIB1 is a pseudokinase that has been implicated in the development of leukemia and also metabolic disorders. It is hypothesized that Trib1 plays an adapter or scaffold function in signaling pathways, notably in MAPK pathways. Therefore, we planned to understand the functional significance of *TRIB1* overexpression in B cells in SLE. We produced a new knock-in model with B-cell-specific overexpression of *Trib1*. We showed that overexpression of *Trib1* specifically in B cells does not impact B cell development nor induce any development of SLE symptoms in the mice. By contrast, Trib1 has a negative regulatory function on the production of immunoglobulins, notably IgG1, but also on the production of autoantibodies in an induced model. We observed a decrease of Erk activation in BCR-stimulated *Trib1* overexpressing B cells. Finally, we searched for Trib1 partners in B cells by proteomic analysis in order to explore the regulatory function of Trib1 in B cells. Interestingly, we find an interaction between Trib1 and CD72, a negative regulator of B cells whose deficiency in mice leads to the development of autoimmunity. In conclusion, the overexpression of *Trib1* could be one of the molecular pathways implicated in the negative regulation of B cells during SLE.

## Introduction

Systemic lupus erythematosus (SLE) is a severe and heterogeneous systemic autoimmune disease, mostly affecting women. Patients produce various pathogenic autoantibodies such as antinuclear antibodies (anti double-stranded DNA, anti-chromatin…), which participate in end-organ damages by a variety of mechanisms, notably *via* immune complex-mediated inflammation leading to glomerulonephritis and vasculitis, for example. The majority of human SLE occurs in adult and the usual evolution of the disease in time is characterized by clinical flares interspersed with silent phases of various lengths (1, 2). To date, we have no molecular explanation to the establishment and the maintenance of these clinically silent phases.

Several lines of evidence indicate that B cells are essential to the disease process and could present intrinsic abnormalities (3, 4): (1) B cells produce the autoantibodies; (2) in murine spontaneous models of SLE, B cells are activated before the disease onset, and in humans, autoantibodies are detectable long before the first symptoms (5); (3) murine models of SLE mice devoid of mature B cells no longer develop lupus phenotype (6); (4) it seems that the important role of B cells in lupus could also implicate their function of antigen presentation to CD4 T cells, and/or cytokine secretion (7). Intrinsic B cell abnormalities are illustrated by the fact that (NZBXNZW)F1 B-lineage cells present an enhanced *in vitro* responsiveness to accessory cell-derived signals (8). Most importantly, the disease can be transferred in mice by B cells: immunodeficient SCID mice populated with pre-B cells from (NZBXNZW)F1 mice, but not those populated with pre-B cells from non-autoimmune mice, develop many of the autoimmune symptoms present in (NZBXNZW)F1 mice, suggesting that genetic defects responsible for the development of SLE disease in (NZBXNZW)F1 mice are intrinsic to their B cells (9).

Considering the central role of B cells in SLE, in a previous work, we performed a genome-wide transcriptome analysis of B cells in lupus patients using microarrays, focusing on the remission phase of the disease, in order to avoid gene expression variations linked to B cell activation which accompanies lupus flares (10). We notably identified an underexpression of *CARABIN*, and then defined Carabin as a new regulator of B cell function by functional genomics in new transgenic mouse models (11). In addition, we described an overexpression of *FKBP11*, which leads in mice to B cell tolerance breakdown and initiates plasma cell differentiation, two features of lupus B cells (12).

Our transcriptome analysis also pointed out an overexpres-sion of *TRIB1* in B cells in quiescent SLE patients. Trib1 belongs to the tribbles family of proteins. The *tribbles* gene was first identified in Drosophila (13). In mammals, tribbles family of proteins is composed of three members: Trib1, Trib2, and Trib3, all pseudokina-ses, whose amino acids sequence is very highly conserved between human and mice. Despite high degrees of similarity between human tribbles protein sequences, Trib1, Trib2, and Trib3 show distinct patterns of expression in human tissues and cellular functions, and are linked to different diseases. Trib1 has been notably linked to the development of human myeloid leukemia and to the negative regulation of lipid metabolism and the development of metabolic disorders (14, 15). It is hypothesized that tribbles play an adapter or scaffold function in signaling pathways, notably in MAPKs pathways (13, 16). Indeed, Trib1 interacts with MEK-1 (upstream activator of ERK) and MKK4 (upstream activator of JNK). Overexpression of Trib1 in HeLa and in murine bone marrow (BM) cells enhances the extent and rate of ERK phosphorylation (17, 18) and inhibits AP1 activity, leading notably to a repression of IL8 promoter (17). But it seems that the expression of tribbles is regulated in a cell-dependent manner, thus contributing to the cell-type specificity of MAPK responses (14). Trib1, as the other tribbles proteins, targets protein substrates to the proteasome and controls their E3 ligase-dependent ubiquitination (16). Trib1 is a serine/threonine pseudokinase containing a N-terminal PEST domain, and a central pseudokinase domain, which could position and regulate potential substrates targeting for ubiquitination. The C-terminal domain of Trib1 contains a MAPKK/MEK regulatory motif, which was shown to bind to MEK1 in some cell types, and an ubiquitin E3 ligase-targeting motif, which binds to COP1 (16). Trib1 is highly expressed in BM, peripheral blood leukocytes (with the highest expression in the myeloid compartment), thyroid gland, and pancreas (16, 17). In immune system, Trib1 is known to be critical for the development of M2 macrophages (19) and to interact with Foxp3 in regulatory T cells (20). However, its role in B cells is totally unknown.

After having confirmed *TRIB1* overexpression in B cells in an additional cohort group of quiescent SLE patients, we planned to understand the functional significance of *TRIB1* overexpression in B cells in human SLE. For this purpose, we generated a new knock-in (KI) model with B-cell-specific overexpression of *Trib1*. We showed that overexpression of *Trib1* specifically in B cells does not impact B cell development nor induce any development of SLE symptoms in the mice. By contrast, Trib1 has a negative regulatory function on production of immunoglobulins, notably IgG1, but also on the production of autoantibodies. Finally, we searched for Trib1 partners in B cells by proteomic analysis in order to decipher the mechanisms of regulatory function of Trib1 in B cells. We notably described for the first time in B cells the interaction between Trib1 and COP1, and with CD72, a negative regulator of B cells whose deficiency in mice leads to the development of autoimmunity. In conclusion, the overexpression of *Trib1* could be one of the molecular pathways implicated in the negative regulation of B cells during SLE.

## Materials and Methods

### Patients

The first cohort comprised 17 patients and 9 age- and sex-matched healthy controls. SLE patients fulfilling at least four diagnostic criteria according to the American College of Rheumatology (21) were prospectively included, provided that they were in a quiescent phase of the disease (with a SLEDAI score less than 4) and were receiving minimal treatment [no immunosuppressive drugs and less than 10 mg of prednisone per day if they needed steroids (4 patients)]. 10 patients were treated with hydroxychloroquine. Purified CD19^+^ B cells from 17 patients blood sample (“cohort 1”) and 9 age- and sex-matched controls were subjected to a pangenomic transcriptome analysis (Affymetrix GeneChip human genome U133 plus 2.0) (10). For the second cohort of patients (“cohort 2”), 4 quiescent patients (3 females and 1 male) aging from 25 to 32 years old with the diagnosis of SLE were selected. Only patients with no treatment, or hydroxychloroquine, or steroids less than 20 mg per day and without immunosuppressive treatments in the previous 6 months, at the time of diagnosis, were included. Patients were compared to healthy age- and sex-matched individuals. Mature naive B cells (CD3^−^CD19^+^CD27^−^IgM^+^CD24^low^CD38^low^) were sorted (FACSAria II, BD Biosciences) and cell viability was assessed with DAPI (Sigma-Aldrich), before extraction of RNA for quantitative real-time RT-PCR analysis.

This study was approved by the ethics committee of the “Hôpitaux Universitaires de Strasbourg” and patients gave their written informed consent.

### Mice

Total RNA was extracted from C57BL/6 total splenocytes using RNeasy Kit (Qiagen). cDNA synthesis was done using High Capacity Reverse Transcription Kit (Applied Biosystems). The coding sequence of mTrib1 (1118 pb, NM144549.4) was amplified from cDNA using the following primers: Forward 5′-ATGCGGGTCGGTCCCGTGCG-3′ and Reverse 5′-CTAGCAGAAGAAGGAACTTATGTCACTG-3′. The PCR conditions were as follows: 94°C for 5 min; 35 cycles at 94°C for 30 s, 58°C for 30 s, and 72°C for 1 min. The PCR product was firstly cloned in the pCR2.1 TA cloning vector (Invitrogen) then two Asc1 restriction sites were added by PCR using the following primers: Forward 5′AAGGCGCGCCGCGCAGATCCAGGGATTTACAAAGCCGGGGCCGCTCCGGCCAGGGCCGCGATGCGGGTCGGTCCC-3′ and Reverse 5′-AAAGGCGCGCCCTAGCAGAAGAAGGA-3′. The PCR conditions were as follows: 94°C for 5 min; 30 cycles at 94°C for 30 s, 60°C for 30 s, and 72°C for 45 s. After a digestion step with AscI (Biolabs), the PCR product was cloned into the CTV Vector (Addgene) (22, 23). The Trib1-ROSA KI mutant mouse line was established at the MCI/ICS (Mouse Clinical Institute—Institut Clinique de la Souris, Illkirch, France^1^). The linearized construct was electroporated in C57BL/6N mouse embryonic stem (ES) cells (ICS proprietary line). After G418 selection, targeted clones were identified by long-range PCR and further confirmed by Southern blot with an internal (Neo) probe and a 5′ external probe. Two positive ES clones were validated by karyotype spreading and microinjected into BALB/C blastocysts. Resulting male chimeras were bred with wild-type C57BL/6N females. Germline transmission was achieved in the first litter. The presence of the transgene in the mice was assessed by a PCR performed on tail DNA, using the following primers: Forward 5′-ACGACCAAGTGACAGCAATG-3′ and Reverse 5′-CTCGACCAGTTTAGTTACCC-3′. Trib1-ROSA Mb1Cre mice were obtained by crossing Trib1-ROSA KI mice with Mb1Cre Mice (24) that will allow *Trib1* overexpression specifically in B cells from the pro-B cell stage. The presence of the Mb1Cre transgene was assessed by a PCR performed on tail DNA, using the following primers: Forward 5′ ACCTCTGATGAAGTCAGGAAGAAC-3′ and Reverse 5′-GCAGATGTCCTTCACTCTGATTCT-3′. All animal experiments were performed with the approval of the “Direction départementale des services vétérinaires” (Strasbourg, France) and protocols were approved by the ethics committee (“Comité d’éthique en matière d’Experimentation Animale de Strasbourg,” CREMEAS, approval number AL/02/15/09/11 and AL/31/38/02/13).

### Quantitative Real-time RT-PCR Analysis

RNA was prepared with RNeasy Kit (Qiagen) and cDNA was obtained with High Capacity Reverse Transcription Kit (Applied Biosystems). For RNA isolated from patients’ cells, a preamplification of 10 ng of cDNA was performed, with TaqMan^®^ PreAmp Master Mix Kit (Applied Biosystems) on a T100™ Thermal cycler (Biorad). Quantitative real-time PCR was performed on 10 ng cDNA using Taqman Universal Mastermix (Applied Biosystems) and Assays-on-Demand probes (Applied Biosystems) (Hprt1: Mm01318743_m1, Trib1: Mm00457875_m1, Pax5: Mm00435501_m1, Blimp1: Mm01187285_m1, total Xbp1: Mm00457357_m1, Bach2: Mm00464379_m1, Bcl-6: Mm00477633_m1, Irf4: Mm00516431_m1, Aicda: Mm00507771_m1, HPRT1: Hs01003267_m1, ACTB: Hs99999903_m1, GAPDH: Hs99999905_m1, TRIB1: Hs00179769_m1). Each sample was amplified in triplicate in a StepOnePlus real-time PCR machine (Applied Biosystems). Relative expression levels were calculated with the StepOne v2.1 software (Applied Biosystems), using the comparative cycle threshold method, and normalized to the endogenous control *Hprt1, Gapdh* and/or *Beta-actin*.

### Flow Cytometry Analysis

Analyses of GFP expression, of cell phenotype, and class-switch recombination were performed on splenic, lymph nodes (LNs), thymic, and BM lymphoid populations by four-color fluorescence analysis according to standard protocols. The following antibodies were used: PE, PerCP, Cy5 or APC anti-mouse B220, CD3, CD4, CD8, CD19, CD21, CD23, CD5, CD86, I-A/I-E, CD44, IgM, IgG1, IgG3, and CD138 (all from BD Biosciences). Propidium iodide was used for discrimination of live and dead cells. For proliferation analysis, cells were permeabilized after extracellular staining and fixed with the cytofix/cytoperm permeabilization kit (BD Biosciences), then stained with the PerCP Cy5.5 anti-Ki67 antibody (BD Biosciences). For intracellular IgG1 staining, membrane B220 staining was performed along with a saturation step using goat anti-mouse IgG (5 µg/mL, Jackson Immunoresearch) for 30 min at 4°C. After a washing step, cells were fixed with 100 µL of fixation buffer from Fixation/Permeabilization kit (eBioscience) during 20 min at room temperature, in the dark. Cells were then permeabilized with the permeabilization buffer from Fixation/Permeabilization kit (eBioscience) and incubated for 30 min at room temperature in the dark with anti-mouse IgG1 (PE, Southern Biotech) and washed before acquisition by a cytometer. For all stainings, cells were analyzed using the FACS Calibur (BD Biosciences). The data were analyzed with FlowJo software (Treestar).

### Mice Immunization

3-month-old mice were injected intraperitoneally at days 0, 7, and 14 with, respectively, 50, 25, and 25 µg of LPS from *S. typhimurium* (Sigma) diluted in PBS; at days 0, 10, and 20 with 100 µg of Ovalbumin (OVA) (Sigma) associated with 250 µg of alum hydroxide; or at days 0 and 23 with, respectively, 100 and 10 µg of NP-KLH (Biosearch technology) associated with 250 µg of alum hydroxide.

### Antibody Detection by ELISA

Total IgG, IgG1, IgG2b, IgG3, or IgM levels were measured in serum from 3- or 6-month-old mice, and in supernatant after 3 days of stimulation, as previously described (12). Anti-dsDNA autoantibodies were measured as previously described (12). Anti-OVA and anti-NP-specific antibodies were measured as previously described (11).

### Cell Preparation and Culture

To evaluate *Trib1* overexpression within the different cell compartments, splenic mature CD43^−^ B cells, CD43^+^ splenocytes and splenic T cells were purified, using B-cell isolation kit (anti-CD43 (Ly-48) microbeads, Miltenyi Biotech) and Dynabeads Untouched Mouse T cells kit (Invitrogen) according to the supplier’s protocols. To study the activation of splenic mature B cells *in vitro*, total splenocytes were plated at 1.10^6^ cells/ml in a culture medium composed of RPMI-1640 (Lonza) containing 10% (v/v) FCS (PAN), 50 mM β-Mercaptoethanol (Gibco), 1% Penicillin/Streptomycin (Gibco), 10 mM HEPES (Lonza), and 1 mM Sodium Pyruvate (Lonza). Cells were stimulated with a combination of LPS (10 µg/mL, Sigma) and IL-4 (10 ng/mL, Sigma), or a combination of IL-4 (5 ng/mL, Sigma), IL-21 (10 ng/mL, Sigma), and anti-CD40 (10 µg/mL, BD Biosciences), or with agonists of TLR1/2 (250 ng/mL, PAM3CSK4, Invivogen), or TLR7 (1 µg/mL, Imiquimod, Invivogen) or TLR9 (5 µM, ODN2395, Invivogen). In some experiments, purified splenic mature B cells (CD43^−^) were plated at 1.10^6^ cells/ml and were stimulated for 3 or 4 days with a combination of LPS (10 µg/mL, Sigma) and IL-4 (10 ng/mL, Sigma) or with LPS only (10 µg/mL, Sigma) using the same culture medium described above for total splenic cells. For the analysis of IgG secretion after blockade of protein transport, GolgiStop™ (BD Biosciences), containing monensin, was added in the appropriate wells directly into the medium (final dilution of GolgiStop™: 1:1,000), 8 h before the acquisition with the cytometer.

To evaluate the activation of signaling pathways by immunoblot analysis, splenic sorted mature B cells were stimulated with F(ab′)_2_ goat anti-mouse IgM antibody at 10 µg/mL (Jackson Immunoresearch).

### Trib1-Flag Expression

Flag-tagged Trib1 and Flag-tagged GFP (negative control) were cloned using standard molecular biology into the pMX-PIE and the pQCXIP retroviral vectors respectively, then were used to establish stably expressing CH12F3 (CH12) cell lines (25) as described previously (26, 27).

### Immunoblot Analysis

Immunoprecipitation (IP) products or protein extracts were loaded on a bisacrylamide gel. Primary antibodies and dilutions were as follows: rabbit anti-phospho Erk (Cell signaling, 1:1,000), rabbit anti-Total Erk (Cell signaling, 1:1,000), anti-phospho Syk (Cell signaling, 1:500), anti-Total Syk (Cell signaling, 1:1,000), mouse anti-phospho IkB (Cell signaling, 1:1,000), rabbit anti-Total IkB (Cell signaling, 1:1,000), mouse anti-Flag-M2 (Sigma, 1/1,000 or 1:20,000), anti-COP1 (Bethyl, 1:1,000), anti-CD72 (Santa Cruz Biotechnology, 1:1,000). The secondary antibodies and dilution were as follows: donkey anti-rabbit IgG (GE Healthcare, 1:10,000), mouse anti-rabbit light chain (Abcam, 1:20,000). The ratio phospho-p42/Total-p42 for one sample corresponds with the ratio between the values of phospho-Erk and total-Erk band density for that sample. The density of each band was measured with ImageJ software.

### Anti-Flag IP and Mass Spectrometry Analysis

Cytoplasmic extracts from CH12-Trib1 and control cell lines were prepared using standard techniques. 20 mg of clarified extracts was taken into IP buffer [IP-300: 20 mM Tris, pH 7.9, 20% glycerol, 300 mM KCl, 0.125 mM EGTA, 0.25 mM EDTA, 1 mM DTT, 0.5 mM PMSF, 1× protease inhibitor cocktail (Roche), 100 U/ml Benzonase (EMD), 0.025% NP-40], and precleared with protein G-agarose beads and mouse IgG (GE Healthcare) for 1 h at 4°C. 40 µL of Flag M2 agarose beads were added (50% slurry; Sigma-Aldrich) and incubated overnight at 4°C. Proteins were eluted three times with 40 µL of Flag peptide (0.2 mg/mL; 30 min at 4°C). Eluted proteins were submitted to identification by mass spectrometry as previously described (27). Proteomics data are available in Table S2 in Supplementary Material.

### Statistical Analysis

Statistical significance was calculated with a two-tailed Mann and Whitney test using Prism software (GraphPad). All data were presented as mean ± SD.

## Results

### Overexpression of TRIB1 in B Cells in SLE Patients

**Figure 1:**
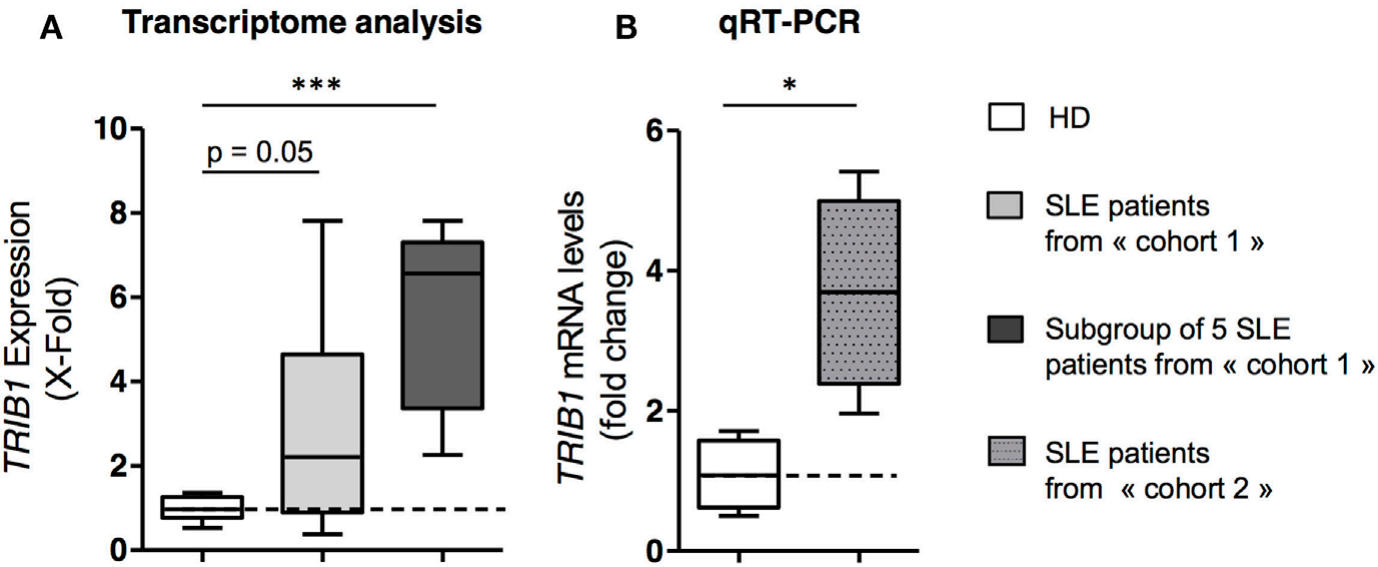
*TRIB1* is overexpressed in B cells from quiescent systemic lupus erythematosus (SLE) patients. **(A)** *TRIB1* mRNA expression levels in transcriptome analysis of purified mature B cells from 17 SLE quiescent patients (light gray) and from a subgroup of 5 patients (dark gray) compared to 9 age- and sex-matched healthy donors (HD). X-fold represents the 2exp^(Pi-Tmean)^ value for the patients, where *Pi* is the value of the TRIB1 probe set signal for a given patient, and *T mean* the mean value of signals for the same probe set for the HD (*T*_mean_ = 8.005). **(B)** Quantitative real-time RT-PCR analysis of *TRIB1* mRNA expression performed on purified B cells from HDs (*n* = 4) and quiescent SLE patients (*n* = 4). Relative expression levels were calculated with the comparative Ct method using the mean of the Ct between *HPRT1, ACTB*, and *GAPDH* for normalization. Each bar represents the level of *TRIB1* mRNA relative to HD controls (Error bars, SD; *p < 0.05, ***p < 0.001, Mann and Whitney test).

We previously analyzed a pangenomic transcriptome of purified CD19^+^ peripheral B cells in patients with inactive SLE in comparison to B cells from age- and sex-matched controls (10). We pointed out a 2.8-times overexpression of *TRIB1* in all patients (*p* = 0.049) (Figure 1A). The overexpression of *TRIB1* was much higher (mean of 5.6-fold over healthy controls) in a subgroup of five patients (Figure 1A) displaying a similar and distinct gene expression pattern with many genes implicated in the unfolded protein response. This subgroup of five patients was not different from the other patients of the same cohort (cohort 1), considering their clinical or phenotypical characteristics (10). TRIB1 overexpression was validated by quantitative real-time RT-PCR in a second cohort of SLE patients versus controls (Figure 1B).

### Generation of B Cell-Specific Trib1 KI Conditional Mice

**Figure 2:**
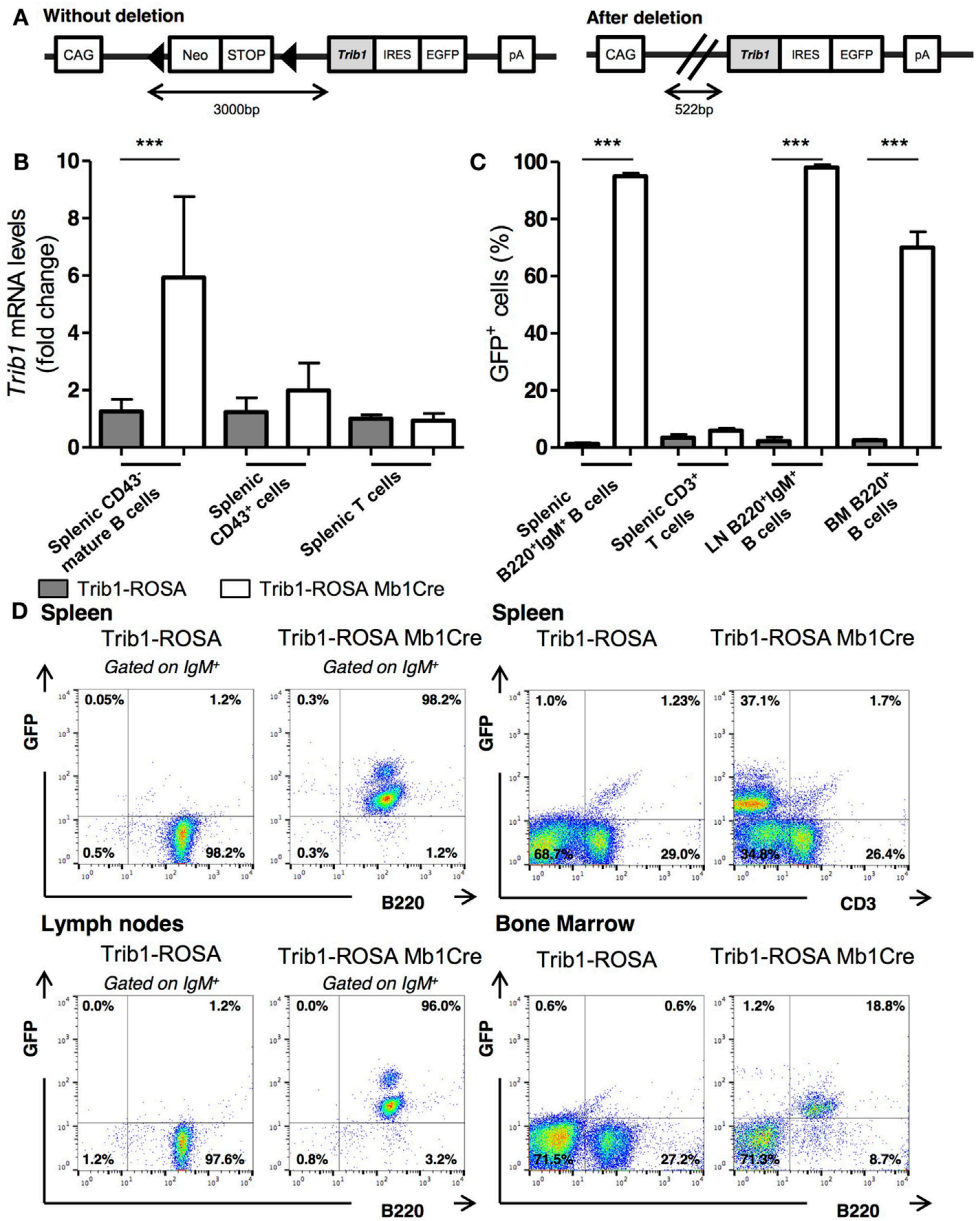
Generation of Trib1-ROSA mice and validation of *Trib1* overexpression and GFP expression in Trib1-ROSA Mb1Cre mice. **(A)** Simplified representation of Trib1-ROSA construct. **(B)** Quantitative real-time RT-PCR analysis of *Trib1* mRNA expression performed on sorted splenic CD43^−^ mature B cells (*n* = 9), splenic CD43^+^ cells (*n* = 9) and splenic T cells (*n* = 3), from 3-month-old Trib1-ROSA and Trib1-ROSA Mb1Cre mice. Each sample was normalized to the endogenous control *Hprt1*. Each bar represents the level of *Trib1* mRNA relative to control mice. **(C)** Percentages of GFP^+^ cells analyzed by flow cytometry **(D)** within B cell or T cell compartment from spleen [B220^+^IgM^+^ B cells (*n* = 9), CD3^+^ T cells (*n* = 4)], LN [B220^+^IgM^+^ B cells (*n* = 9)], and BM [B220^+^ B cells (*n* = 9)] of Trib1-ROSA and Trib1-ROSA Mb1Cre mice (Error bars, SD; ***p < 0.001, Mann and Whitney test).

As the role of Trib1 in B cells and in autoimmunity has never been described, we decided to investigate in detail the possible role of TRIB1 overexpression in B cell function and in the promotion of SLE. Therefore, we developed a functional genomic study in mice, by the generation of B cell-specific Trib1 KI conditional mice. We inserted the coding sequence for murine *trib1*, preceded by the synthetic CAG promoter and a loxP-flanked Neo-STOP cassette, into the ubiquitously expressed ROSA26 locus in the genome of C57BL/6 mice. We used the CTV vector designed by Xiao et al. in Rajewsky’s lab (22). A frt-flanked IRES-EGFP cassette, which is placed between the cloning site for *trib1* insertion and the polyadenylation signal (pA), allows the detection of cells in which excision of Neo-STOP cassette has been efficient and, therefore, constitutes a good reporter for *Trib1* overexpression (Figure 2A). The mice obtained by this strategy were named Trib1-ROSA mice. These Trib1-ROSA mice were used as controls in all experiments. Then, we developed a B cell-specific Trib1 KI model, by crossing Trib1-ROSA mice with Mb1 Cre animals. The *mb-1* gene encodes the BCR Ig-α subunit (CD79a), and is expressed from the very early pro-B cell stage in BM (24). The mice, overexpressing *Trib1* specifically in B cell lineage, will be thereafter named Trib1-ROSA Mb1Cre.

We first evaluated the specificity of *trib1* overexpression in B cells in Trib1-ROSA Mb1Cre mice. We detected a six-times overexpression of *Trib1* mRNA in sorted splenic mature B cells in Trib1-ROSA Mb1Cre mice, compared to control mice, by quantitative real-time RT-PCR. The overexpression was not seen in the rest of splenic cells, neither in sorted splenic T cells (Figure 2B). We also analyzed the expression of GFP, reporter for Trib1 overexpression, in different lymphoid organs by flow cytometry. The average percentage of GFP^+^ cells was very high in B220^+^IgM^+^ B cells in spleen and LNs of Trib1-ROSA Mb1Cre mice (98.0 and 95.0%, respectively), compared to control mice (1.4 and 2.3%, respectively), with almost no GFP expression in splenic CD3^+^ T cells (5.8 and 3.5% in Trib1-ROSA Mb1Cre and control mice, respectively) (Figures 2C,D). These results show that the deletion of the STOP cassette was very efficient and specific of the B cell lineage in Trib1-ROSA Mb1Cre mice. Finally, about 70% of BM B220^+^ cells express GFP, compared to about 1% in control mice (Figures 2C,D). This could be explained in part by the fact that, during B cell development in BM, B220 is expressed earlier (at Hardy’s fraction A, i.e., pre-proB stage) than Ig-α (Hardy’s fraction B, i.e., proB cell stage) (28). In conclusion, as expected with the Mb1Cre model, *Trib1* is specifically overexpressed in B cell lineage in Trib1-ROSA Mb1Cre mice.

### B Cell Phenotype in Mice Overexpressing *Trib1* in B Cells

We next assessed the development of B cells in primary and secondary lymphoid organs in Trib1-ROSA Mb1Cre mice, by flow cytometry analysis. The proportions of the different subsets of B cells in BM, spleen, and LNs were not different between Trib1-ROSA Mb1Cre and Trib1-ROSA control mice, showing that *Trib1* overexpression in B cells does not have any impact on their development (Table 1). We detected in the spleen and LNs two distinct populations of GFP positive B cells in Trib1-ROSA Mb1Cre mice (Figure 2D). However, the proportions of MZ, T1, T2, and follicular B cells were not different between splenic GFP^+^ and GFP^high^ populations. Of note, as expected, *Trib1* overexpression in B cells did not impact neither T cell development (Table S1 in Supplementary Material), nor the expression of activation markers CD44 and CD69 after stimulation with anti-CD3 and anti-CD28 antibodies for 72 h *in vitro* (data not shown). Because SLE is often associated with B cell hyper-activity (2), we analyzed the expression of activation markers (CD86, MHC II, CD44) on B cells in spleen and LNs. The results showed that *Trib1* overexpression in B cells does not increase basal activation of B cells in secondary lymphoid organs (Figure S1 in Supplementary Material). In addition, the expression of CD86 expression on splenic B cells was not different between Trib1-ROSA Mb1Cre and Trib1-ROSA mice, after *in vitro* stimulation with various TLR agonists (Figure S2 in Supplementary Material).

**Table 1:** Flow cytometry analysis of B cell subsets from bone marrow (BM), spleen, and lymph nodes (LN) does not show any defect in B-cell development and differentiation in Trib1-ROSA Mb1Cre mice.

	Trib1-ROSA (*n* = 9)	Trib1-ROSA Mb1Cre (*n* = 9)
**BM**		
Pro-pre B	8.3 ± 2.2%	8.0 ± 2.9%
Immature	1.9 ± 1.0%	1.7 ± 0.7%
Transitional	1.4 ± 0.7%	0.8 ± 0.4%
Mature	1.8 ± 1.0%	1.5 ± 0.6%
**Spleen**		
Total cellularity	*66.6 10*^^6^^ ± *2.0.10*^^7^^	*60.7 10^6^* ± *1.9.10^7^*
Total splenic B cells	39.2 ± 12.7%	46.0 ± 10.3%
	*25.4 10^6^* ± *10.1 10^6^*	*28.2 10^6^* ± *12.0 10^6^*
Fo	29.9 ± 11.2%	34.1 ± 5.1%
	*17.1 10^6^* ± *6.0 10^6^*	*20.6 10^6^* ± *6.8 10^6^*
MZ	1.3 ± 0.5%	2.5 ± 2.5%
	*0.9 10^6^* ± *0.6 10^6^*	*1.8 10^6^* ± *2.4 10^6^*
T1	10.8 ± 4.1%	10.3 ± 1.8%
	*6.4 10^6^* ± *2.1 10^6^*	*6.4 10^6^* ± *2.8 10^6^*
T2	7.3 ± 2.9%	6.4 ± 1.8%
	*4.2 10^6^* ± *1.9 10^6^*	*3.9 10^6^* ± *1.8 10^6^*
PB	1.1 ± 1.0%	1.1 ± 1.0%
	*0.74 10^6^* ± *0.68 10^6^*	*0.67 10^6^* ± *0.68 10^6^*
B1 cells	0.8 ± 0.7%	0.7 ± 0.4%
	*0.43 10^6^* ± *0.37 10^6^*	*0.9 10^6^* ± *0.8 10^6^*
**LN**		
Total cellularity	*6.3 10*^6^ ± *4.2 106*	*5.1 10^6^* ± *3.0 10^6^*
Total LN B cells	28.1 ± 11.4%	28.0 ± 4.0%
	*2.2 10^6^* ± *1.8 10^6^*	*1.5 10^6^* ± *1.1 10^6^*
PB	1.7 ± 0.7%	1.9 ± 0.3%
	*0.09 10^6^* ± *0.06 10^6^*	*0.11 10^6^* ± *0.05 10^6^*

*BM: Pro-Pre B (B220^+^IgM^−^); Immature (B220^int^IgM^+^); Transitional (B220^int^IgM^high^); Mature (B220^high^IgM^+^). Spleen/LN: Fo: Follicular (B220^+^IgM^+^CD23^+^CD21^low^); MZ: Marginal Zone (B220^+^IgM^+^CD23^−^CD21^high^); T1: Transitional 1 (B220^+^IgM^+^CD23^−^CD21^−^); T2: Transitional 2 (B220^+^IgM^+^CD23^+^CD21^high^); PB: Plasmablast (B220^+^CD138^low^); B1 cells (CD19^+^B220^+^CD5^+^)*.

*italic: absolute numbers*.

**Figure 3:**
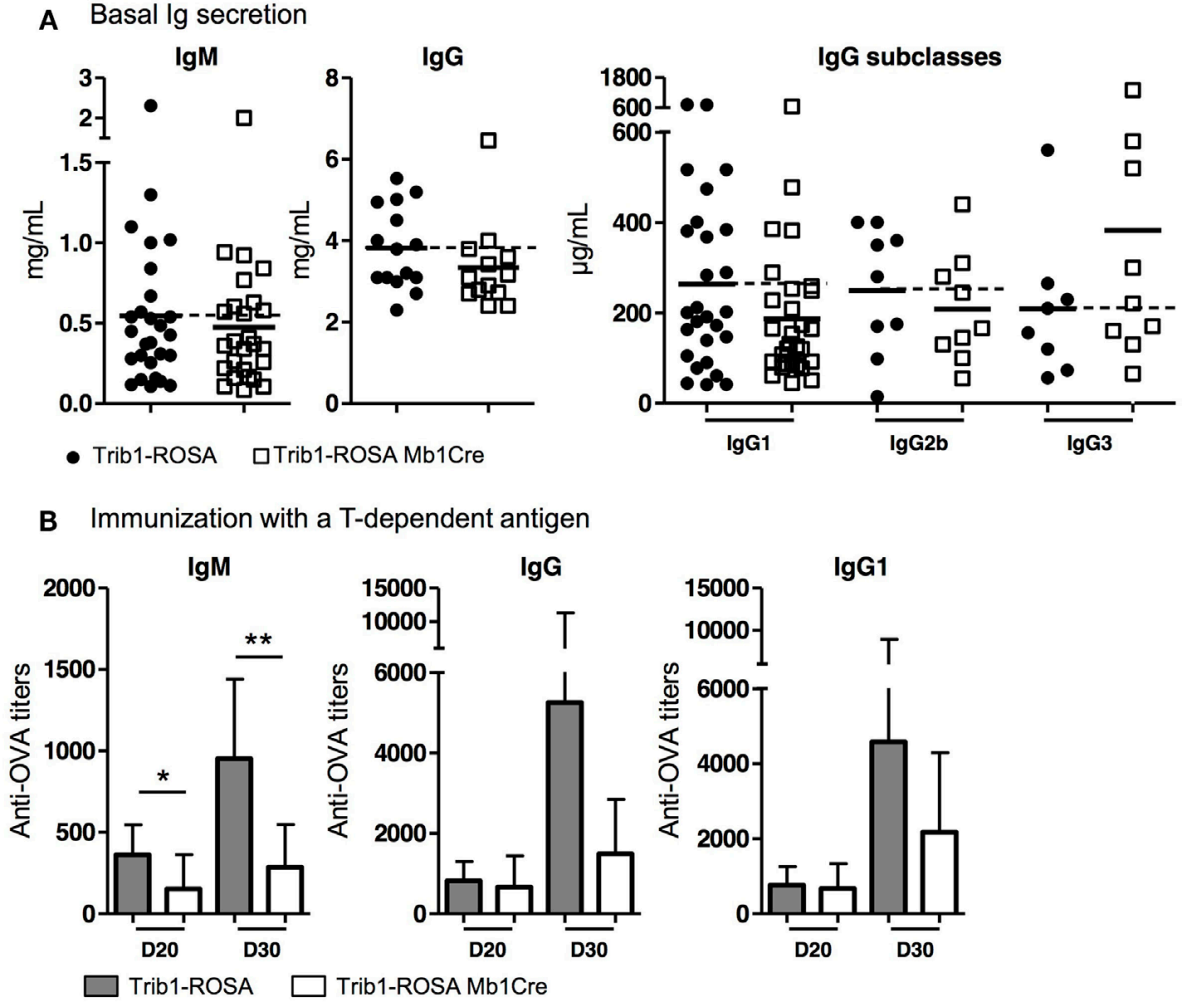
Ig production in Trib1-ROSA Mb1Cre compared to Trib1-ROSA control mice, at basal level and after immunization with a T-dependent antigen. **(A)** Sera from 6-month-old Trib1-ROSA and Trib1-ROSA Mb1Cre mice were collected and total IgM and IgG, IgG1, IgG2b, and IgG3 concentrations were determined by ELISA. Each dot represents the result for one animal. **(B)** Trib1-ROSA mice (*n* = 7) and Trib1-ROSA Mb1Cre mice (*n* = 6) were immunized with ovalbumin (OVA) associated with alum hydroxide at days 0, 10, and 20. Serum was collected at days 20 and 30, then anti-OVA IgM, IgG, and IgG1 titers were measured by ELISA (*p < 0.05, **p < 0.001, Mann and Whitney test).

A majority of SLE patients and lupus murine models develop a hypergammaglobulinemia (2, 29). The level of serum IgM was comparable between the two groups of mice, and the levels of IgG was even decreased in Trib1-ROSA Mb1Cre mice compared to Trib1-ROSA mice, although the difference was not statistically significant (Figure 3A). When IgG subclasses were quantified in serum, we noticed that the most decreased IgG subclass was IgG1 (186 µg/mL in Trib1-ROSA Mb1Cre versus 263.5 µg/mL in control mice) (Figure 3A). The production of antigen-specific antibodies was decreased after immunization with a T-dependent antigen (OVA) in Trib1-ROSA Mb1Cre mice compared to control mice, at day 20 for IgM and at day 30 for IgM and IgG, and the decrease was statistically significant for IgM (Figure 3B).

In conclusion, *Trib1* overexpression in B cells does not have any impact on the development of B cells, nor on their activation status, but seems to have a slight negative impact on immunoglobulin production.

### B-Cell-Specific Trib1 Overexpressing Mice Do Not Develop Any Sign of Lupus

**Figure 4:**
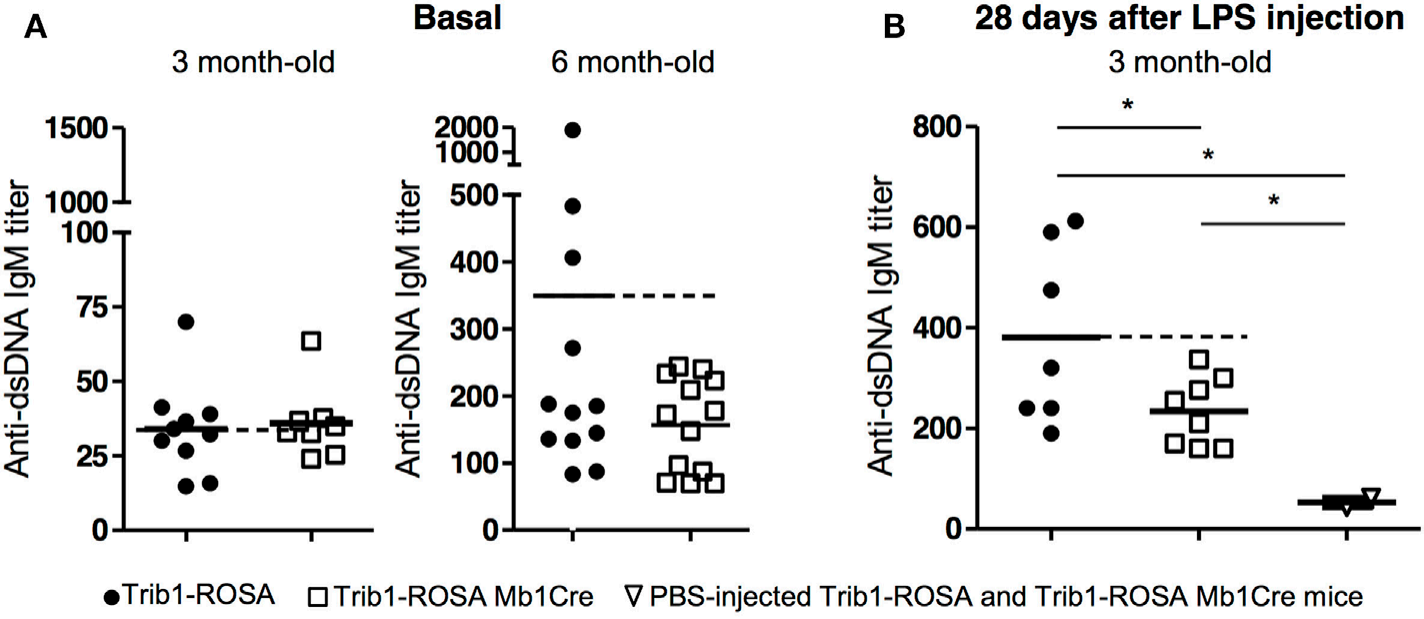
*Trib1* overexpression in B cells induces a decrease of autoantibody production in a LPS-induced lupus mouse model. **(A)** Sera from 3-month-old and 6-month-old Trib1-ROSA and Trib1-ROSA Mb1Cre mice were collected and basal anti-dsDNA IgM autoantibody titers were determined by ELISA. **(B)** 3-month-old Trib1-ROSA and Trib1-ROSA Mb1Cre mice were injected with PBS or LPS (at days 0, 7, and 14) and bleeded every week until day 28. The titers of anti-dsDNA IgM at day 28 were measured by ELISA. Because results were not different between PBS-injected Trib1-ROSA and Trib1-ROSA Mb1Cre mice, these mice were pooled on the PBS-injected control group. Each dot represents the result for one animal (*p < 0.05, Mann and Whitney test).

Trib1-ROSA Mb1Cre mice were analyzed to evaluate the development of lupus symptoms. They do not develop any proteinuria, even at old age (18 months) (data not shown). We quantified the basal level of serum anti-dsDNA IgM autoantibodies, one of the hallmarks of SLE disease, by ELISA. At a young age (3-month-old), Trib1-ROSA Mb1Cre and Trib1-ROSA produce the same amount of anti-dsDNA IgM, whereas at 6 months, Trib1-ROSA Mb1Cre mice produce even less autoantibodies than control mice (Figure 4A). At 3 months, as mice from C57BL/6 genetic background do not produce high titers of autoantibodies, we boosted the production of anti-dsDNA autoantibodies by an injection of LPS. The injection of LPS mimics a bacterial infection and induces a polyclonal activation of B cells, including autoreactive B cells producing natural autoantibodies. LPS from *Salmonella typhimurium* was chosen because it was shown to induce a high production of anti-DNA antibodies in young C57BL/6 and in (NZB × NZW)F1 mice (30). Mice were injected at days 0, 14, and 28, and the production of anti-dsDNA IgM was quantified at day 28 by ELISA. The injection of LPS induced an increase of anti-dsDNA IgM production both in Trib1-ROSA Mb1Cre and in Trib1-ROSA control mice compared to PBS-injected mice (Figure 4B). However, B-cell-specific *Trib1* overexpressing mice produce less anti-dsDNA IgM than control mice, and the difference was statistically significant. In conclusion, the overexpression of *Trib1* in B cells does not induce the development of SLE. On the contrary, it could have a regulatory function on anti-dsDNA antibody production, considering the results obtained *in vivo* in an induced model of autoantibody production.

### *Trib1* Overexpression in B Cells Negatively Regulates Ig Production

**Figure 5:**
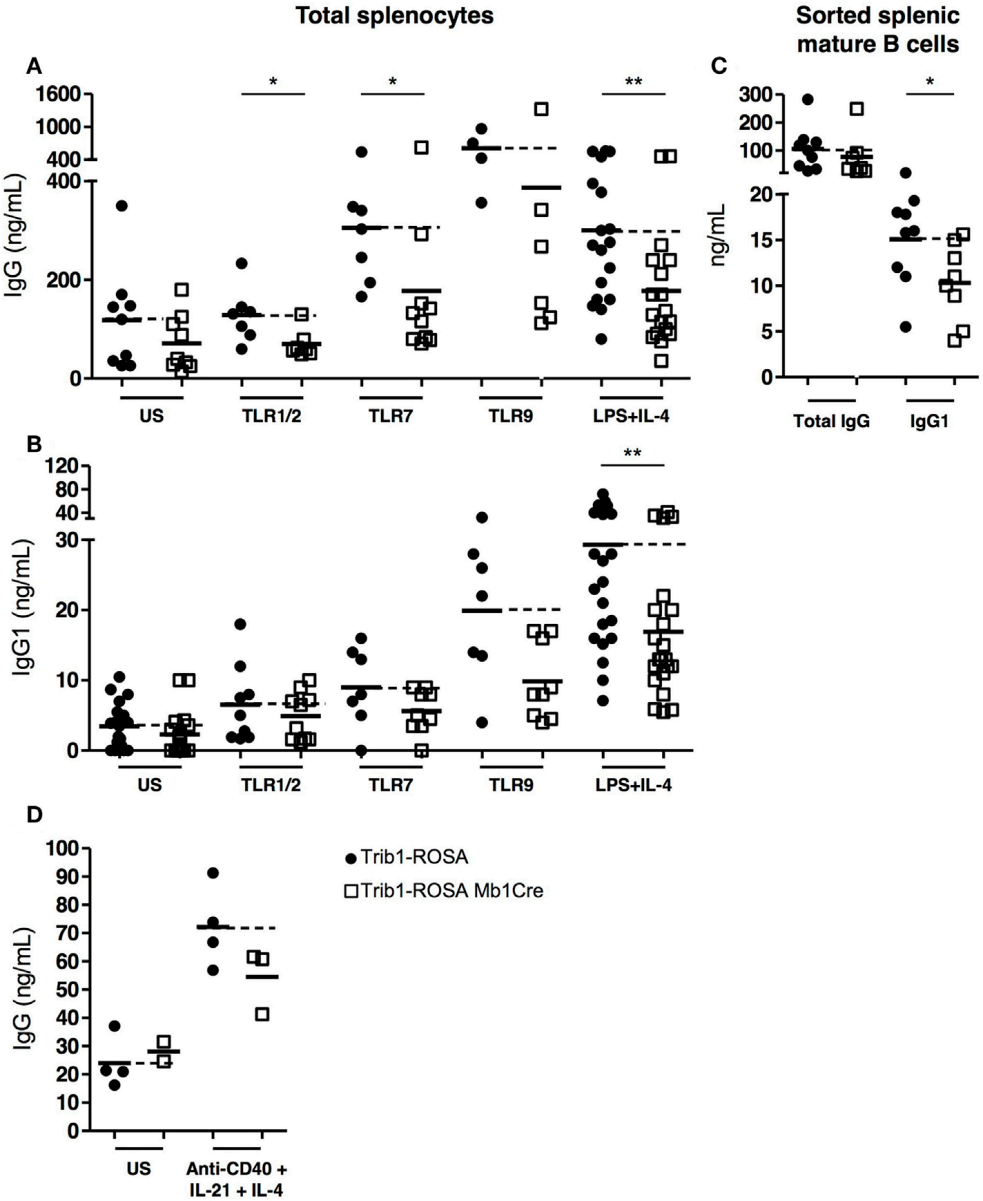
*Trib1* overexpression in B cells negatively regulates Ig production *in vitro*. **(A,B)** Total splenocytes were collected from Trib1-ROSA and Trib1-ROSA Mb1Cre mice and stimulated with TLR ligands or a combination of LPS (TLR4 ligand) and IL-4 during 72 h *in vitro*. The concentration of total IgG **(A)** and IgG1 **(B)** in the supernatant was measured by ELISA. **(C)** Splenic mature B cells were purified from Trib1-ROSA and Trib1-ROSA Mb1Cre mice and stimulated *in vitro* with a combination of LPS and IL-4. The concentration of total IgG and IgG1 in the supernatant was measured by ELISA. **(D)** Total splenocytes from Trib1-ROSA and Trib1-ROSA Mb1Cre mice were stimulated with anti-CD40, IL-4, and IL-21 during 72 h *in vitro*. The concentration of total IgG in the supernatant was measured by ELISA. Each dot represents the result for one animal (US: unstimulated) (*p < 0.05, **p < 0.001, Mann and Whitney test).

In order to better understand the impact of *Trib1* overexpression on Ig (and notably IgG1) production, we stimulated total splenocytes and splenic sorted B cells with various stimuli, including a combination of LPS and IL-4, known to induce the class-switching of B cells into IgG1-producing cells, and with several TLR agonists (TLR7, TLR9, and TLR1/2) known to play a role in lupus physiopathology in both lupus mouse models and patients (31). We stimulated total splenocytes from Trib1-ROSA Mb1Cre and Trib1-ROSA control mice during 72 h with TLR agonists or with a combination of LPS and IL-4 and quantified the production of total IgG and IgG1 in supernatants by ELISA. All tested stimuli induced a decrease of IgG and IgG1 secretion in culture supernatants from Trib1-ROSA Mb1Cre splenocytes, compared to Trib1-ROSA splenocytes. The decrease in IgG production was notably statistically significant after TLR1/2 and TLR7 agonists stimulation (Figures 5A,B). The stimulation of total splenocytes with LPS (TLR4 agonist) alone led to a non-statistically significant decrease of IgG secretion in Trib1-ROSA Mb1Cre mice, compared to Trib1-ROSA mice (data not shown), however, LPS in combination with IL-4 induced a statistically significant decrease of both IgG and IgG1 secretion (Figures 5A,B). In both cases, IgM secretion was not affected (data not shown). Importantly, this defect of IgG/IgG1 secretion was intrinsic to B cells, because the stimulation of sorted splenic mature B cells only, with LPS and IL-4, led also to a decrease of IgG1 production (Figure 5C). We also tested a stimulation protocol that better mimics T cell help: the stimulation of total splenocytes with anti-CD40, IL-4, and IL-21 induced a non-statistically significant decrease of IgG secretion in Trib1-ROSA Mb1Cre mice, compared to Trib1-ROSA mice (Figure 5D), and IgM secretion was marginally affected (data not shown).

**Figure 6:**
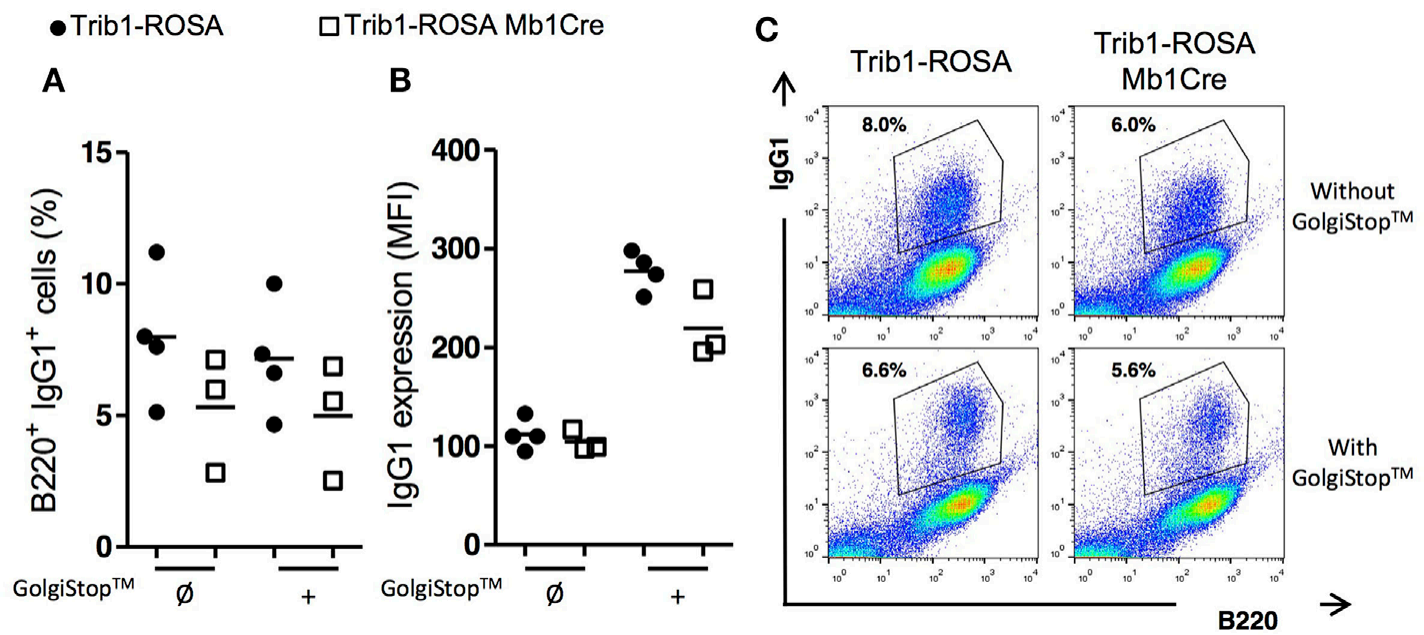
Trib1 overexpression in B cells induces a reduction in the production of secreted form of IgG1. Total splenocytes from Trib1-ROSA and Trib1-ROSA Mb1Cre mice were stimulated with LPS/IL-4 for 72 h *in vitro* [with (+) or without (Ø) the addition of a protein transport inhibitor “GolgiStop™” for the last 8 h of culture], then stained for intracytoplasmic IgG1 after a step of membrane Ig blocking using an anti-murine IgG antibody, a step of fixation and permeabilization, and finally were analyzed by flow cytometry. **(A)** Percentage of B220^+^ B cells stained for intracellular IgG1. **(B)** MFI of intracellular IgG1 staining on B220^+^ B cells. **(C)** A representative sample of each condition is shown. Each dot represents the result for one animal.

Then we analyzed the potential mechanisms leading to Ig production defect in *Trib1* overexpressing B cells. As mentioned earlier, this deficiency could not be attributed to a defect in B cell activation process, considering the equal/similar expression of activation markers after stimulation *in vitro* (Figure S2 in Supplementary Material). An increase of B cell mortality or a decrease of B cell proliferation could be responsible for the phenotype. However, there was no difference between the two groups of mice in the percentages of dead cells (Figures S3A,B in Supplementary Material) nor in the percentage of proliferating (B220^+^Ki67^+^) B cells after stimulation *in vitro* (Figures S3C,D in Supplementary Material). We also analyzed the plasma cell (PC) differentiation process. We tested two known models of *in vitro* plasmablast differentiation, i.e., stimulation of B cells with LPS and with LPS/IL-4, then we quantified the percentage of B220^low^CD138^+^ plasmablasts by flow cytometry. Our results showed no difference between the two groups of mice (Figures S4A,B in Supplementary Material). In addition, we analyzed the expression of plasma cell differentiation program genes (repressed genes, such as *Pax5, Bach2, Bcl-6, Aicda*; induced genes, such as *Blimp1, Xbp1*, and *Irf4*) by quantitative real-time RT-PCR analysis. Considering the results, *Trib1* overexpression does not interfere with PC differentiation genetic program (Figures S4C,D in Supplementary Material). Finally, a defect of class-switching could lead to a decrease of Ig secretion by *Trib1* overexpressing B cells. We stimulated total splenocytes with LPS and IL-4 (for IgG1 class-switching) and with LPS (for IgG3) and quantified the MFI for surface Ig expression and the percentages of IgG1^+^ or IgG3^+^B220^+^ cells by flow cytometry. We did not detect any difference between *Trib1* overexpressing and control B cells (Figures S5A–C in Supplementary Material). The blockade of protein transport with GolgiStop™, containing monensin, in the culture of splenocytes with LPS and IL-4, showed a decrease of intracellular IgG1 positive B cells and of intracellular IgG1 MFI in B cells, in Trib1-ROSA Mb1Cre compared to control mice (Figure 6). In conclusion, *Trib1* overexpression in B cells negatively regulates the Ig secretory capacity.

### Trib1 Interacts with CD72 and Its Overexpression Affects Erk Signaling

**Figure 7:**
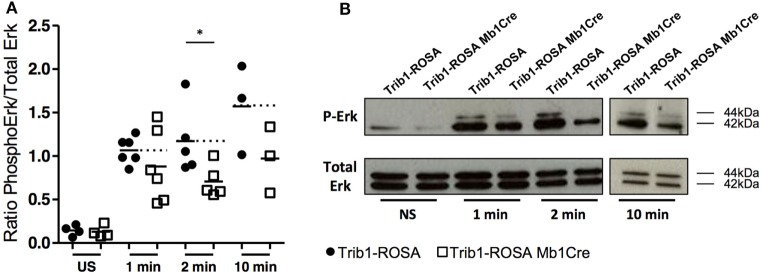
*Trib1* overexpression in B cells induces a decrease of Erk phosphorylation after BCR engagement. Purified mature splenic B cells (CD43^−^) from Trib1-ROSA and Trib1-ROSA Mb1Cre mice were stimulated with anti-IgM (10 µg/mL) for the indicated time. Cell lysates were analyzed by western blot using anti-phospho Erk antibody (P-Erk). Total Erk was used as a loading control. The ratio of phospho-p42/Total p42 was calculated for each condition and represented in **(A)**. Each dot is representative of one mouse. Representative immunoblots of the indicated stimulation timepoints are presented in **(B)**. (US: unstimulated) (*p < 0.05, Mann and Whitney test).

In order to find hypotheses on the mechanisms of B cell phenotype due to *Trib1* overexpression, and considering the role of Trib1 in MAPK signaling (13, 16), we analyzed Erk signaling in splenic sorted mature B cells from Trib1-ROSA-Mb1 and control mice, after stimulation with anti-IgM antibody, by Western Blot. In-deed, Erk-dependent pathway is one of the major MAPK pathways activated in B cells after BCR engagement. The phosphorylation of Erk was decreased in B cells from Trib1-ROSA-Mb1 mice, compared to control mice, and the difference was statistically significant at 2 min of stimulation (Figure 7). Proximal BCR signaling Syk pathway was not affected (data not shown). The defect in Erk pathway in *Trib1* overexpressing B cells probably does not explain the phenotype of these cells on its own. Therefore, we determined the partners of Trib1 in B cells in order to have a better insight of Trib1 function in these cells. We transduced a murine B cell line (CH12) with a retrovirus encoding a Flag-mTrib1 and IRES-GFP reporter (CH12-Trib1) or only a Flag-GFP as a control (CH12-GFP). To identify cytoplasmic partners of Trib1, we performed Flag IP followed by SDS-PAGE and mass spectrometry identification.

**Figure 8:**
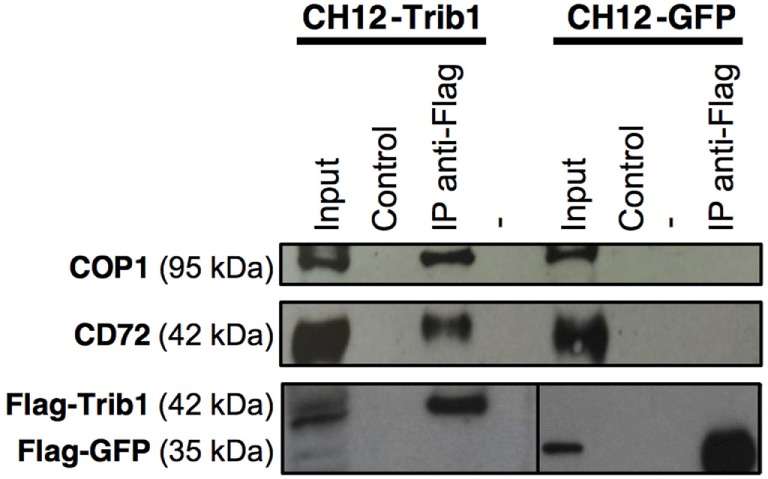
Trib1 interacts with COP1 and CD72 in B cells. CH12 cells were transfected with pMX-PIE/Flag-tagged Trib1 or GFP. The samples were immunoprecipitated with an anti-Flag antibody to precipitate both Flag-tagged proteins from cell cytoplasmic lysates (IP anti-Flag) or with total mouse serum (Control). The result is representative of at least three independent experiments. Note: considering the differences in Flag protein expression in CH12-Trib1 and CH12-GFP cells, two different dilutions of anti-Flag antibody were used for the bottom left part (1:1,000) and the bottom right part (1:20,000). (Input, total protein lysate; -, empty well).

Mass spectrometry analysis of 5 independent experiments revealed a list of 236 proteins specifically enriched in CH12-TRIB1 cells. Proteins were ranked according to the number of experiments in which they were identified, and their confidence score. We kept those proteins identified in at least three out of the five experiments (Table 2). These proteins were classified into the following groups: proteins implicated in ubiquitination process, regulators of signal transduction, regulators of protein production, and secretion and chaperone proteins (Table 2). Among the previously identified and best characterized partner of Trib1, COP1 (13, 32), we found additional proteins, including notably CD72 (Table 2). CD72 is a very interesting candidate because it is a well-known negative B cell regulator. The association between Trib1, COP1, and CD72 was verified by co-IP and Western Blot (Figure 8). Several partners of Trib1 are linked to the regulation of signal transduction (for example Spre1, Stk40, and Ppm1b) (Table 2; Figure S6 in Supplementary Material). Interestingly, Spre1 has been described as a negative regulator of Erk pathway (33, 34). Further experiments will be needed to understand the potential implication of these partners in the negative regulatory function of Trib1 in B cells.

**Table 2:** Proteins implicated in protein ubiquitination, signal transduction, protein production secretion are found as Trib1 partners in CH12 B cell line.

Accession number	Full name	Function	*n*	Score
**Protein ubiquitination**				
COP1	E3 Ubiquitin-protein ligase COP1	Ubiquitin ligase	5	112
MALT1	Isoform 2 of mucosa-associated lymphoid tissue lymphoma translocation protein 1	Ubiquitin ligase—involved in BCL-10-induced NF-κB pathway activation	3	16

**Signalization and signal transduction**				
Stk40	Serine/threonine-protein kinase 40	Serine/threonine kinase, negative regulator of NF-κB pathway, and p53-induced gene transcription	5	45
St38L	Serine/threonine-protein kinase 38-like	Kinase—involved in signal transduction regulation	4	45
MARCS	Myristoylated alanine-rich C-kinase substrate	Protein kinase C substrate	4	19
LAP2	Isoform 2 of Protein LAP2	HER2 adaptor—Inhibitor of NOD2-dependant NF-κB pathway and pro-inflammatory cytokine secretion	3	71
PPM1B	Isoform Beta-2 of protein phosphatase 1B	Associated to IKKB dephosphorylation and inactivation (NF-κB pathway)	3	23
CALM	Calmoduline	Implicated in calcium flux	3	21
FLII	Protein flightless-1 homolog	Involved in estrogen-induced signaling	3	18
CD72	B-cell differentiation antigen CD72	Negative regulator of B cells	3	16
SPRE1	Sprouty-related, EVH1 domain-containing protein 1	MAPK pathway regulator	3	15
JAK3	Tyrosine-protein kinase JAK3	Kinase involved in cytokine production pathway, cell development, differentiation, and proliferation	3	10
TR19L	Tumor necrosis factor receptor superfamily member 19 L	Involved in NF-κB pathway activation	3	8

**Protein production and secretion**				
Eif4b	Eukaryotic translation initiation factor 4B	Required for mRNA binding to ribosome	3	39
EPN4	Clathrin interactor 1	Associated to clathrin vesicles transport from Trans-Golgi to endosomes	3	30
RS6	40S ribosomal protein S6	Ribosomal protein	3	15
RS18	40S ribosomal protein S18	Ribosomal protein	3	13
STX7	Syntaxin-7	Implicated in protein traffic from early endosomes to plasma membrane	3	13
RS19	*40S ribosomal protein S19*	Ribosomal protein	3	7

**Chaperone protein**				
HS90B	Heat shock protein HSP 90-beta	Chaperone protein	4	37
HS90A	Heat shock protein HSP 90-alpha	Chaperone protein	4	29
CDC37	Hsp90 co-chaperone Cdc37	Chaperone protein	3	14

**Others**				
RNF219	RING Finger protein 219	Zinc finger protein	5	122

*Mass spectrometry analysis was performed on immunoprecipitation product (n_total_ = 5). In each subgroup defining their main function, the proteins are ranked according to the number of experiments in which they were found as Trib1 partners (column « n ») and their confidence score. (Accession numbers are coming from Swissprot Database)*.

## Discussion

We identified an overexpression of *TRIB1* in human SLE B cells during clinically inactive disease by transcriptome analysis (10). This overexpression was confirmed in a second cohort of SLE quiescent patients, by real-time qRT-PCR. This led us to study the consequences of *Trib1* overexpression on B cell phenotype and on the development of SLE in a new murine transgenic model. For this purpose, we developed a B-cell-specific Trib1 KI conditional model, by an insertion of murine *Trib1* coding sequence, preceded by a floxed STOP cassette, into ROSA locus. We detected a six-times overexpression of *Trib1* in splenic mature B cells in this model, thanks to an efficient deletion of the STOP cassette by the Cre recombinase under the control of Mb1 promoter. Therefore, the level of *Trib1* overexpression in this model was close to the one detected in B cells from quiescent SLE patients in our transcriptome analysis or in the second cohort of patients. Because the overexpression of *Trib1* begins at very early stage in B cell lineage in BM, we checked if *Trib1* overexpression in our model could have an impact on B cell development. Our results showed that this was not the case. Altogether, these characteristics made this new mouse model suitable for this study.

Trib1-ROSA-Mb1 mice did not display any symptom of lupus disease: activation status of B cells is normal, and we did not detect any feature of renal disease. On the contrary, we showed that *Trib1* overexpression specifically in B cells had a negative impact on autoantibody production, notably after induction of anti-dsDNA antibody production with injection of LPS, mimicking an infectious event. Moreover, *Trib1* overexpression in B cells negatively regulated Ig production, *in vivo* at basal level or after immunization notably with a T-dependent antigen, and also after *in vitro* stimulation. The most impacted Ig subtype was IgG1. However, the effect does not seem to be specific of IgG1. Because class switching into IgG subclasses is notably guided by specific stimuli (as LPS and IL-4 in murine B cells, for IgG1) (35), one could hypothesize that the degree of engagement of Trib1 in this negative regulation depends on the signaling pathways activated in B cells and, therefore, on the receptor engaged at the surface of the cells. Our results showed that the defect in Ig production was neither the consequence of an activation defect of B cells, nor an increase of mortality or a decrease of viability, nor a defect in class switching and plasma cell differentiation program. However, we detected a decrease of the percentage of intracellular IgG1 positive B cells and of intracellular IgG1 MFI in B cells in Trib1-ROSA Mb1Cre compared to control mice after stimulation with LPS and IL-4. In conclusion, it seems that the decrease in Ig levels was mostly the consequence of a defect in Ig secretory capacity. Interestingly, the analysis of *Trib1* expression in the different B cell subpopulations has been done in C57BL/6 mice (corresponding to background of our Trib1 transgenic model),^2^ and in humans notably for B cells and plasma cells^3^ and shows that *Trib1* is highly expressed in plasmablasts and in plasma cells, thereby arguing for an important function of Trib1 in Ig production.

We searched for Trib1 partners in murine B cells, in order to understand the immunosuppressive role of Trib1. After IP of Trib1 in CH12 B cell line and mass spectrometry identification, we selected a short list of Trib1 partners that could potentially be implicated in signal transduction and protein production and secretion. Among these partners, COP1 E3 ubiquitin ligase was already identified as a partner of Trib1 in different cell types (13, 19, 32), but never in B cells. It was notably shown in myeloid cells that COP1, via Trib1, targets C/EBPα for degradation by the proteasome (32). In mammals, C/EBPα is an important transcription factor controlling myeloid differentiation (13). According to this function, it is almost exclusively expressed in myeloid cells^4^; therefore, this could explain why we did not identify it as a partner of Trib1 in B cells. We confirmed the association of COP1 with Trib1 in CH12 cells by IP and Western-Blot analysis. It is known that COP1 binding to Trib1 is essential to target protein substrates for degradation (13). Further experiments should explore in details the role of COP1 in the degradation of proteins implicated in Ig synthesis and secretion in B cells. Moreover, we pointed out JAK3 as a Trib1 partner in B cells. JAK3 is a kinase associated with different cytokine receptors, notably IL-4 receptor, therefore giving one explanation for the phenotype of Trib1 overexpressing B cells after stimulation with LPS and IL-4.

The regulation of several signaling pathways was a frequent characteristic of the partners we identified for Trib1 in B cells (Table 2). Trib1 has been mostly described in the literature as a regulator of MAPK signaling, in different cell types. In HeLa cells, BM and myeloid leukemia cells, Trib1 was shown to interact, *via* its C-terminus domain, with MEK-1, and to be responsible for a hyperphosphorylation of Erk (13, 17). In other cell types, as muscular cells, Trib1 overexpression does not have any impact on Erk phosphorylation (36). Generally speaking, Tribbles proteins seem to act either as activators or inhibitors of MAPK pathways (17). We showed here that, in murine B cells, Trib1 overexpression seems to decrease Erk phosphorylation. This could be linked to the defect in Ig production and secretion observed in Trib1-ROSA-Mb1 mice. Among the partners, we identified for Trib1 in B cells, Spred1 is a suppressor of Ras signaling, notably in innate lymphoid cells (34). Therefore, Trib1, *via* the recruitment of Spred1, could negatively regulate Erk signaling in B cells. The immunosuppressive role of Trib1 in B cells probably also implicate other partners. We identified CD72 as another cofactor of Trib1 in B cells. CD72 is a negative regulator of B cells (37). It is a very interesting candidate because the production of immunoglobulin is increased in CD72-deficient mice (38), and a decreased expression of CD72 was associated with an increased surface IgG on B cells and to a severe disease in patients with lupus nephritis (39), arguing for a negative role of CD72 on immunoglobulin production. Moreover, the expression of the “b” isoform of CD72 which is the one expressed in C57BL/6 mice (and, therefore, in the mice analyzed in our study) and in CH12 cells (40), in MRL.CD72^b^/lpr congenic mice, is protective against the development of the lupus disease (41, 42). In addition, CD72 downregulates BCR signaling, and notably NF-κB and Erk (43–45). Our preliminary experiments showed that NF-κB signaling was not affected in *Trib1* overexpressing B cells after stimulation of the BCR pathway (with anti-IgM antibody). However, it could be interesting to see if it is also the case when CD72 and BCR pathways are synergistically activated.

In conclusion, we described a new role of Trib1 as a negative regulator of B cells. Despite the polygenic nature of lupus disease in humans, one feature of B cells from quiescent SLE patients, i.e., Trib1 overexpression, in mice, is sufficient on its own to have an immunosuppressive effect on B cells. It would be interesting to see the effect of B cell-specific *Trib1* overexpression on the development of the disease in an SLE murine model. Moreover, there is no molecular explanation for the phenotype of SLE patients during silent phases of the disease, or for the maintenance of the clinically silent phases (46). As such, the overexpression of TRIB1 in B cells could constitute one of these protective molecular pathways. It would be interesting in the future to analyze the overexpression of *TRIB1* in B cells in a prospective way, in order to study the correlation between *TRIB1* expression and the quiescent or active nature of the disease in the same patient.

## Ethics Statement

This study was carried out in accordance with the recommendations of the ethics committee of the “Hôpitaux Universitaires de Strasbourg” with written informed consent from all subjects. All subjects gave written informed consent in accordance with the Declaration of Helsinki. The protocol was approved by the ethics committee of the “Hôpitaux de Strasbourg” (Strasbourg, France). This study was carried out in accordance with the recommendations of “Direction départementale des services vétérinaires” (Strasbourg, France). Protocols were approved by the ethics committee (“Comité d’éthique en matière d’Experimentation Animale de Strasbourg,” CREMEAS, approval number AL/02/15/09/11 and AL/31/38/02/13).

## Author Contributions

LS, VD, JR-L, TM, A-SK, BRSM, and PS-S designed the research. LS, VD, JR-L, AS, VH, IR, VG, and DB performed the research. LS, VD, JR-L, VH, VG, TM, A-SK, BRSM, and PS-S. analyzed the data. LS, VD, IR, A-SK, BRSM, and PS-S wrote the paper.

## Conflict of Interest Statement

The authors declare that the research was conducted in the absence of any commercial or financial relationships that could be construed as a potential conflict of interest.

## Footnotes

^1^http://www.ics-mci.fr/en/.

^2^https://www.immgen.org.

^3^http://amazonia.transcriptome.eu.

^4^http://immgen.org.

## Supplementary Material

TABLE S1 | T cell subpopulations from Trib1-ROSA and Trib1-ROSA Mb1Cre were analyzed by flow cytometry

TABLE S2 | Proteomics raw data.
