# Supplementary material for: Trib1 Is Overexpressed in Systemic Lupus Erythematosus, While It Regulates Immunoglobulin Production in Murine B Cells
Source: Front Immunol. 2018 Mar 15;9:373. doi: 10.3389/fimmu.2018.00373 (PMC5862796; doi:10.3389/fimmu.2018.00373)
Supplement: TABLE S1 — | T cell subpopulations from Trib1-ROSA and Trib1-ROSA Mb1Cre were analyzed by flow cytometry [file table_1.docx]

|  | **Trib1-ROSA** | **Trib1-ROSA Mb1Cre** |
| --- | --- | --- |
| **Thymus** | (n=3) | (n=3) |
| Total cellularity | 67.0 10^6^ ± 1.9 10^7^ | 73.3 10^6^ ± 2.5 10^7^ |
| CD4^+^ | 7.5% ± 1.7% 5.0 10^6^ ± 1.9 10^6^ | 7.0% ± 0.3% 5.1.10^6^ ±1.5 10^6^ |
| CD8^+^ | 3.5% ± 0.5%  2.2 10^6^ ± 0.4 10^6^ | 3.8% ± 1.6%  2.7 10^6^ ± 1.3 10^6^ |
| CD4^+^ CD8^+^ | 84.5% ± 0.9%  56.7 10^6^ ± 1.6 10^7^ | 83.0% ± 6.9%  61.0 10^6^ ± 2.3 10^7^ |
| CD4^-^ CD8^-^ | 5.7% ± 2.5%  3.6 10^6^ ± 1.2 10^6^ | 5.7% ± 0.8%  4.2 10^6^ ± 1.5 10^6^ |
| **Spleen** | (n=9) | (n=9) |
| Total cellularity | 66.6 10^6^ ± 2.0 10^7^ | 60.7 10^6^ ± 1.9 10^7^ |
| CD4^+^ | 14.4% ± 4.9% 10.5 10^6^ ± 4.3 10^6^ | 13.9% ± 3.0% 8.4 10^6^ ± 3.1 10^6^ |
| CD8^+^ | 11.9% ± 5.1% 8.3 10^6^ ± 3.8 10^6^ | 11.0% ± 2.0% 6.7 10^6^ ± 2.2 10^6^ |
| **Lymph nodes** | (n=9) | (n=9) |
| Total cellularity | 6.3 10^6^ ± 4.2 10^6^ | 5.1 10^6^ ± 3.0 10^6^ |
| CD4^+^ | 30.3% ± 6.2% 1.8 10^6^ ± 1.1 10^6^ | 29.5% ± 3.6% 1.4 10^6^ ± 0.8 10^6^ |
| CD8^+^ | 28.0% ± 4.4% 1.5 10^6^ ± 0.8 10^6^ | 27.3% ± 4.3% 1.3 10^6^ ± 0.6 10^6^ |

Table S1: *Trib1* overexpression does not impact T cell development and differentiation in Trib1-ROSA Mb1Cre mice.
