# Supplementary figures and images for: Trib1 Is Overexpressed in Systemic Lupus Erythematosus, While It Regulates Immunoglobulin Production in Murine B Cells
Source: Front Immunol. 2018 Mar 15;9:373. doi: 10.3389/fimmu.2018.00373 (PMC5862796; doi:10.3389/fimmu.2018.00373)

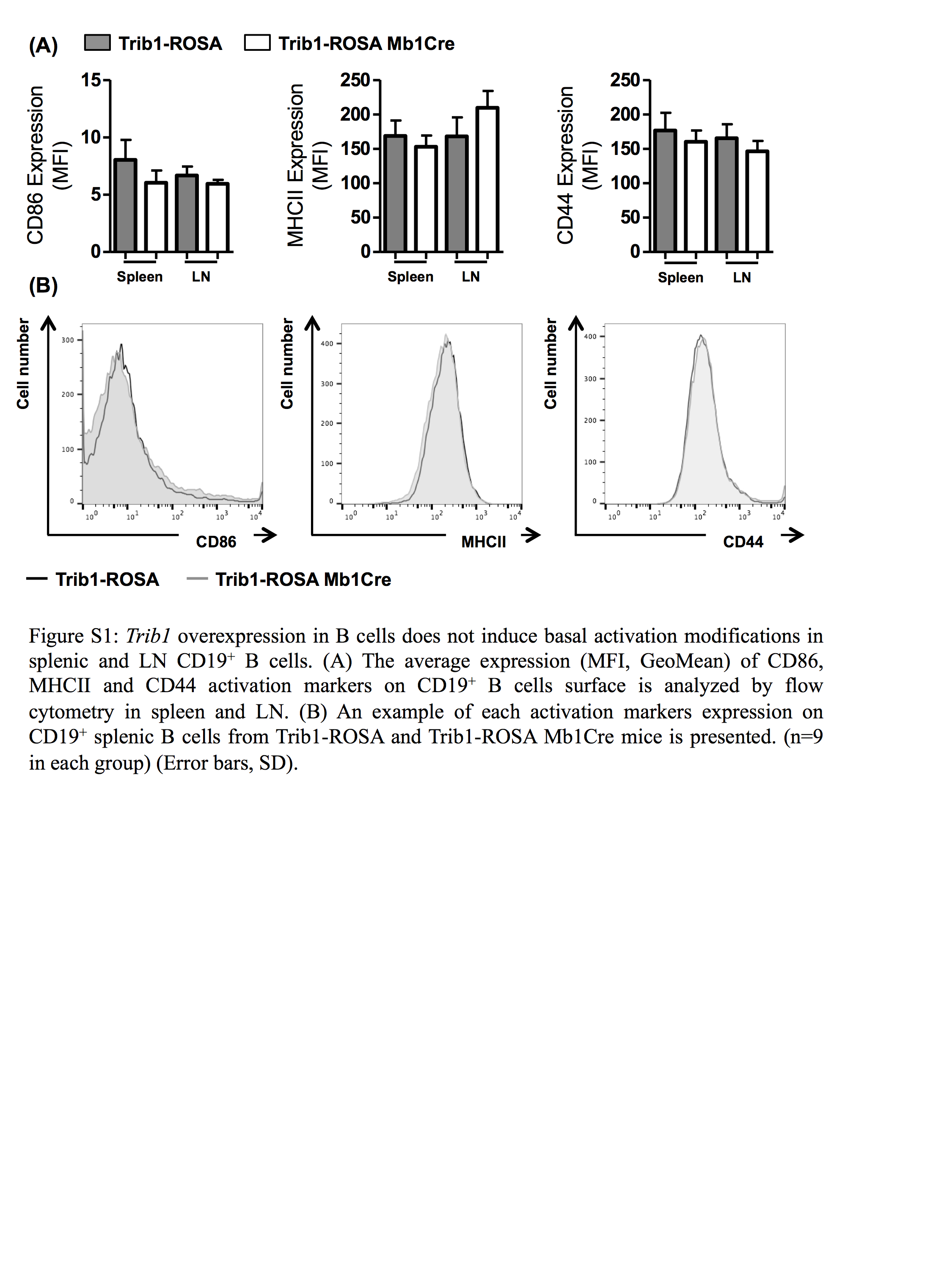

Supplement: Supplementary file 3 [file image_1.jpeg]

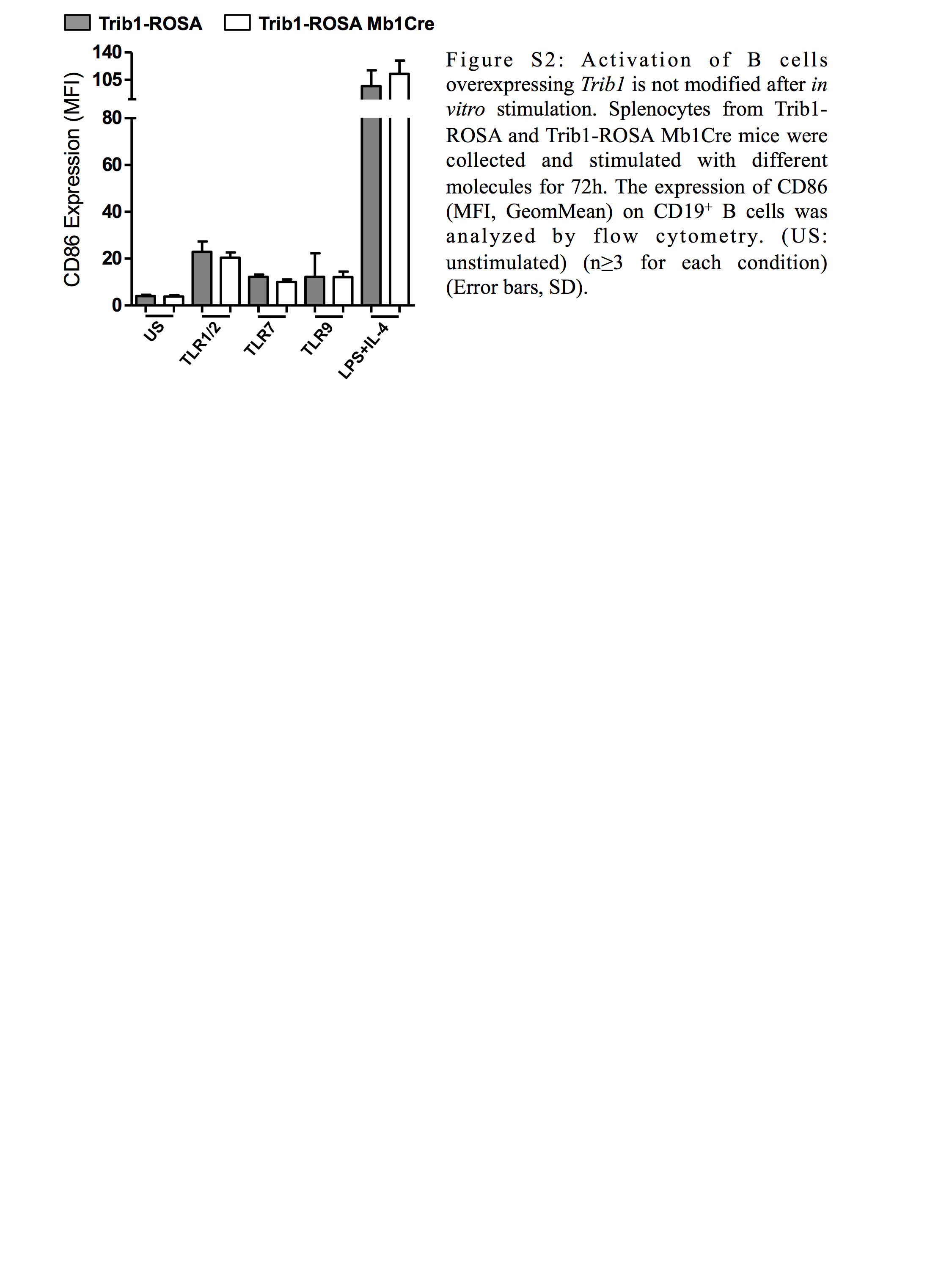

Supplement: Supplementary file 4 [file image_2.jpeg]

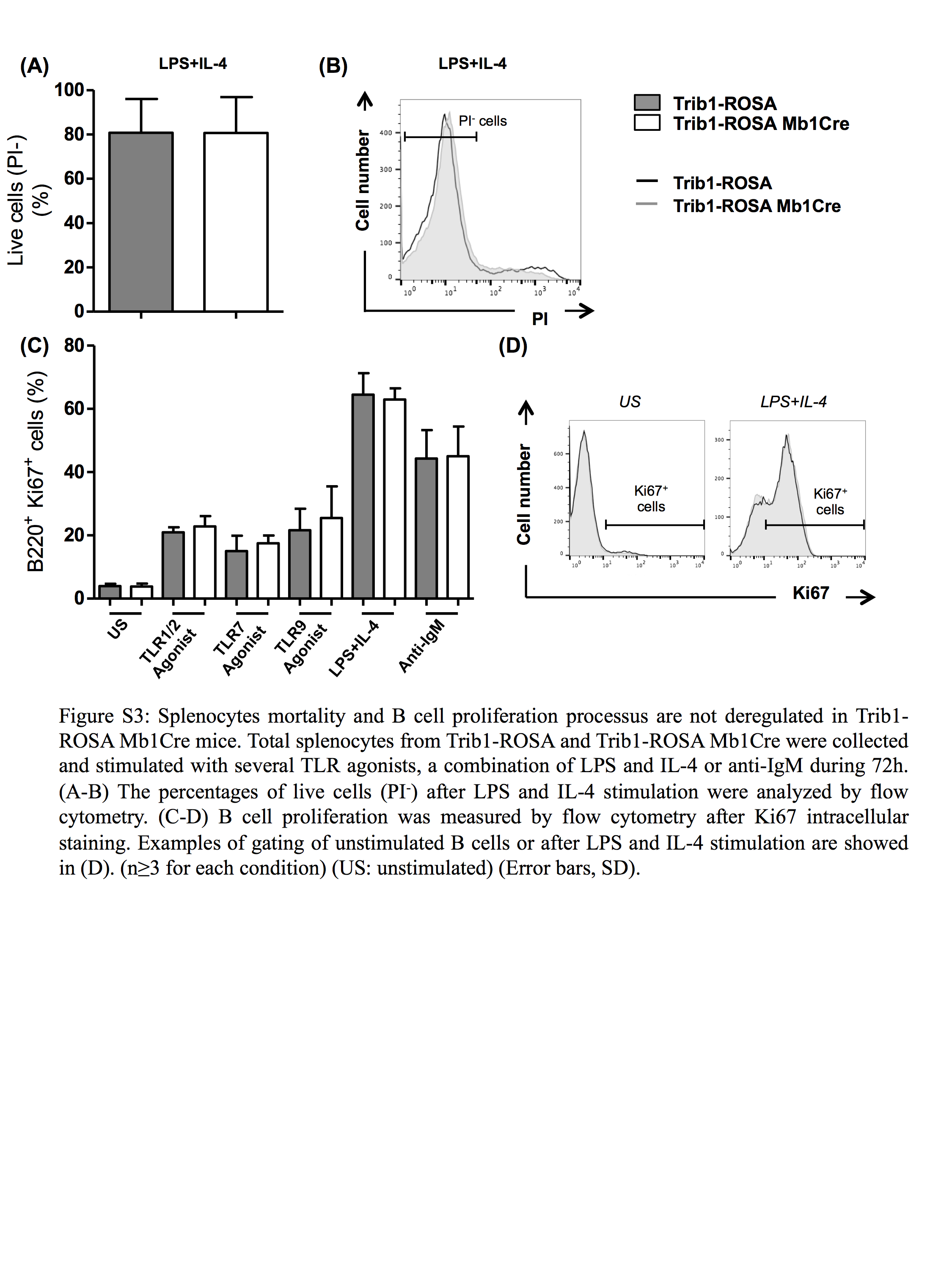

Supplement: Supplementary file 5 [file image_3.jpeg]

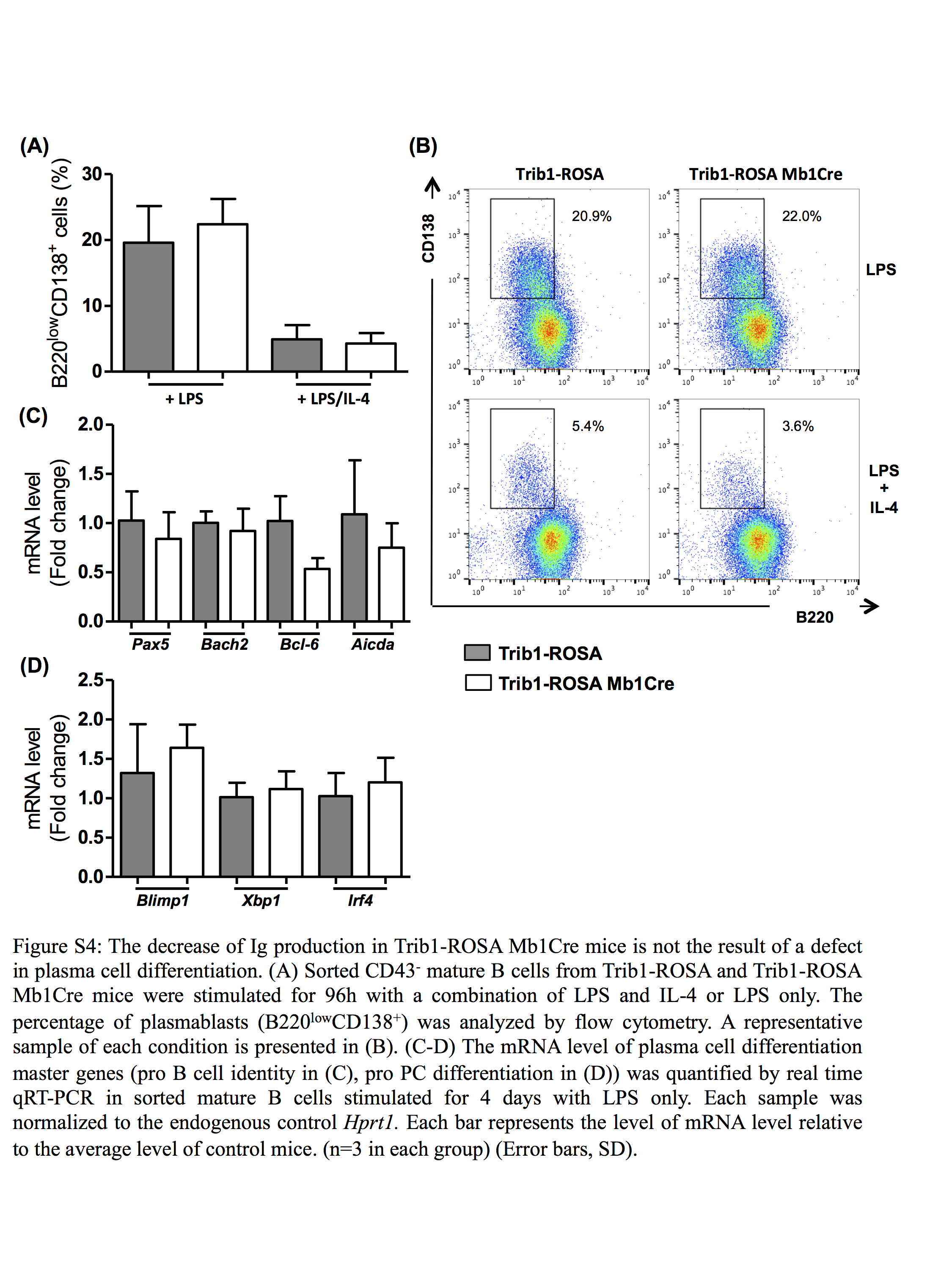

Supplement: Supplementary file 6 [file image_4.jpeg]

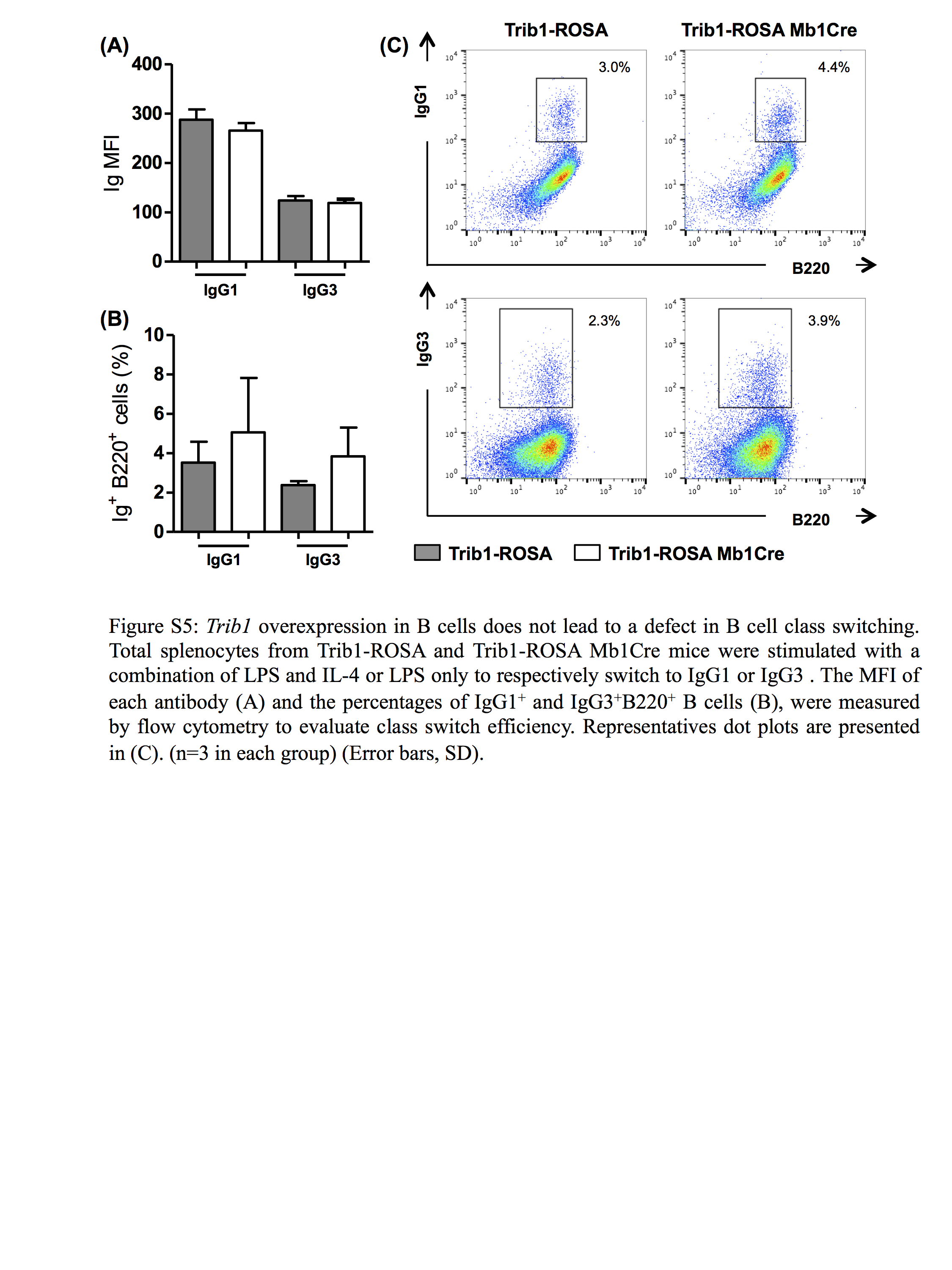

Supplement: Supplementary file 7 [file image_5.jpeg]

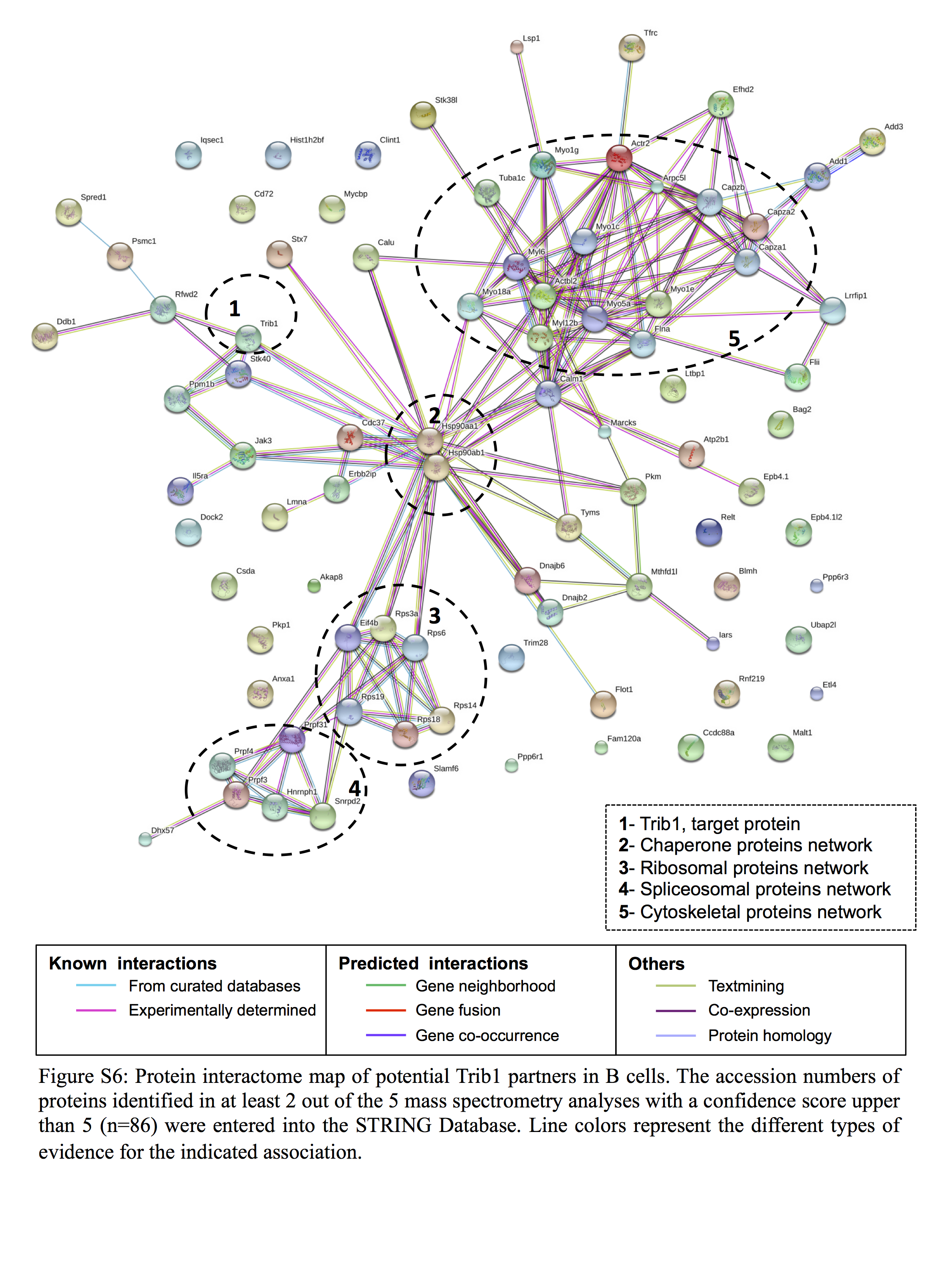

Supplement: Supplementary file 8 [file image_6.jpeg]
